# Photochemistry of the α-Al_2_O_3_-PETN Interface

**DOI:** 10.3390/molecules21030289

**Published:** 2016-02-29

**Authors:** Roman V. Tsyshevsky, Anton Zverev, Anatoly Mitrofanov, Sergey N. Rashkeev, Maija M. Kuklja

**Affiliations:** 1 Department of Materials Science and Engineering, University of Maryland, College Park, MD 20742, USA; rtsyshev@umd.edu (R.V.T.); rashkesn@yahoo.com (S.N.R.); 2 Department of Organic and Physical Chemistry, Kemerovo State University, Kemerovo 650043, Russia; anthon.zverev@yandex.ru (A.Z.); anatoly.y.mitrofanov@gmail.com (A.M.); 3 Yurga Institute of Technology, National Research Tomsk Polytechnic University, Yurga 652057, Russia; 4 Qatar Environment & Energy Research Institute, Hamad Bin Khalifa University, Qatar Foundation, P.O. Box 5825, Doha, Qatar

**Keywords:** singlet-triplet exciton, electronically excited and charged state, decomposition barrier, initiation of detonation, explosives, F-centers, oxygen vacancy

## Abstract

Optical absorption measurements are combined with electronic structure calculations to explore photochemistry of an α-Al_2_O_3_-PETN interface formed by a nitroester (pentaerythritol tetranitrate, PETN, C_5_H_8_N_4_O_12_) and a wide band gap aluminum oxide (α-Al_2_O_3_) substrate. The first principles modeling is used to deconstruct and interpret the α-Al_2_O_3_-PETN absorption spectrum that has distinct peaks attributed to surface F^0^-centers and surface—PETN transitions. We predict the low energy α-Al_2_O_3_ F^0^-center—PETN transition, producing the excited triplet state, and α-Al_2_O_3_ F^0^-center—PETN charge transfer, generating the PETN anion radical. This implies that irradiation by commonly used lasers can easily initiate photodecomposition of both excited and charged PETN at the interface. The feasible mechanism of the photodecomposition is proposed.

## 1. Introduction

With many unresolved research challenges, photo-stimulated processes in composite systems, containing several components, became important objects of study by many researchers in different areas of chemistry, physics, and material science. Applications of photo-processes are compelling but interactions of materials with light are manifested in many different ways and hence are still poorly understood. For example, charge carrier excitations in light-sensitizer (dye) organometallic molecules with subsequent charge transfer to metal oxides are widely studied for developing novel efficient solar cells [1,2,3]. In contrast to photovoltaics, in photocatalysis [1,4], charge carriers formed by absorption of photons remain in the photocatalyst and do not transfer across the interfaces. The ability of the material to change its electro-physical properties (the band gap, electroconductivity, and the type of conductivity) under adsorption of gas molecules is employed for developing solid state gas sensors. Photo-sensitivity and selectivity of nanostructures [5,6], organic molecules [7], metal-organic frameworks [8], and polymers [9,10,11,12] among other systems have been long explored for sensing [13,14], and detecting [15,16,17] of high-power explosives, in order to prevent threats to human security, locate buried land mines, and aid environmental protection efforts.

Our current research was inspired by an idea of achieving a highly controllable explosive decomposition chemistry initiated by photo-excitation with standard available lasers. In a series of recent experiments [18,19,20,21] and quantum-chemical simulations [22,23], it was fundamentally established that the decomposition of a high explosive material, PETN, can be reliably triggered by laser irradiation once PETN crystals are mixed with a small fraction of micron size metal oxide (e.g., MgO) powders. This demonstrated that there is a strong dependence of the initiation process on the presence of the oxide. The PETN and MgO are wide gap dielectrics, which are completely transparent to the laser light of 1.17 eV [20,21]. However, once they form an interface, or a composite, their optical, electronic, and photochemical properties significantly change. Thus, oxygen vacancies at the MgO surface facilitate strong chemical adsorption of PETN on MgO, induce a charge transfer, and consequently may initiate the PETN decomposition by laser light with the photon energy of 1.17 eV, with an unusually low activation barrier [22,23].

To validate and refine the notion of laser initiation of explosive decomposition chemistry in high energy density materials, we explored an α-Al_2_O_3_-PETN composite. We expect that this system is somewhat different from the MgO-PETN composite but it will exhibit similar photo-chemistry and enrich our knowledge of organic crystal—oxide composite systems. Here we report results of the combined theoretical and experimental study of the electronic structure, optical properties, and photodecomposition of α-Al_2_O_3_-PETN composite by Nd:YAG laser irradiation (1.17 and 2.33 eV) in order to understand: (i) how optical absorption of the composite material differs from the properties of the pristine individual PETN and α-Al_2_O_3_ materials; and (ii) how the defect- and interface-induced changes of the electronic structure affect the chemical reactivity of adsorbed molecules. The developed methodology and achieved conclusions will further clarify atomic scale mechanisms of charge transfer on surface defects and interfaces between molecular crystals and oxides thus providing a solid basis for fundamental understanding of decomposition chemistry of highly energetic materials, energy storage and conversion, photocatalysis, and molecular electronics.

## 2. Results and Discussion

### 2.1. Optical Absorption of α-Al_2_O_3_-PETN Composites

#### 2.1.1. Structures of Model Supercells

**Figure 1:**
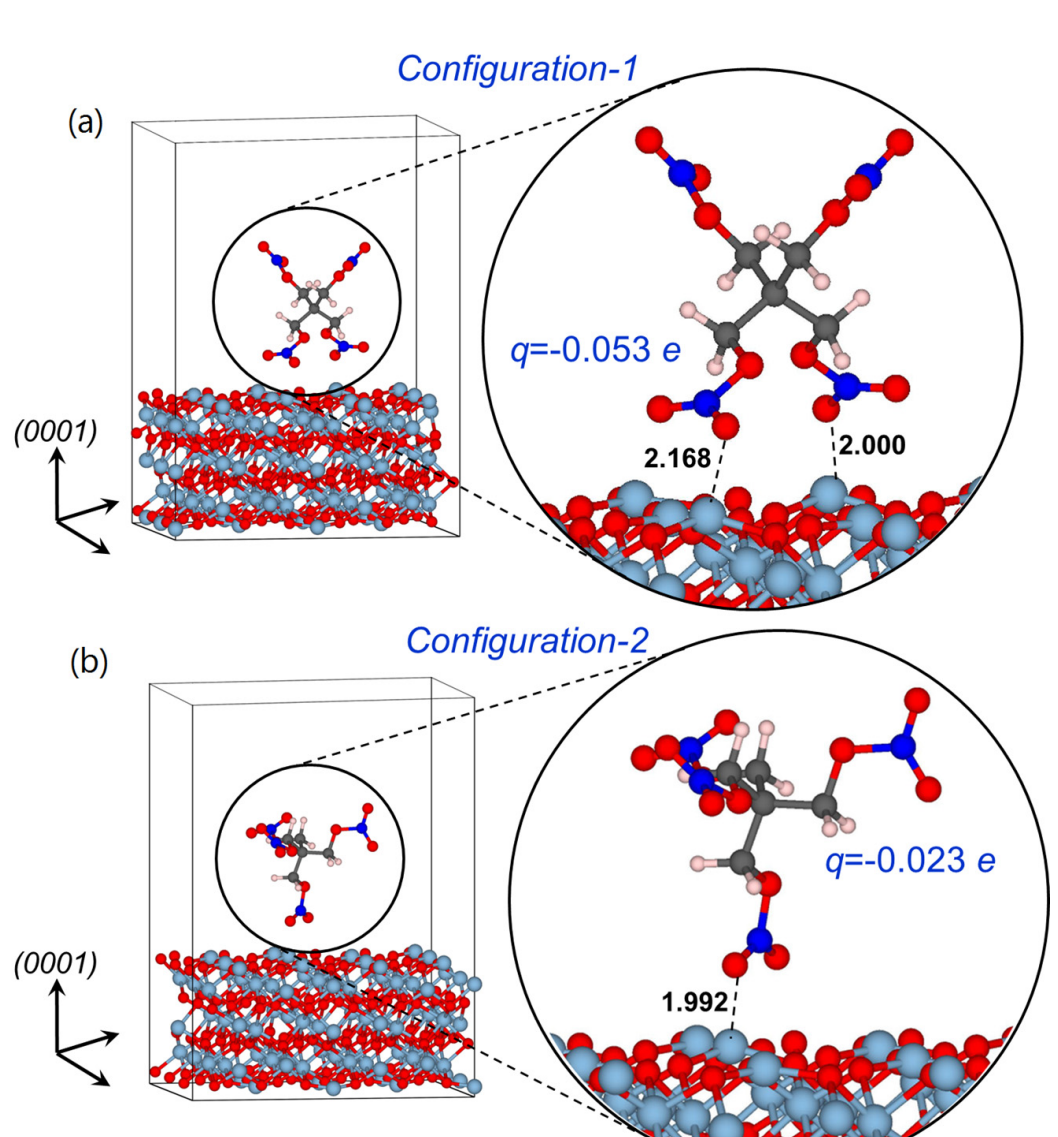
Schematics of the model supercells representing a PETN molecule adsorbed at the (0001) α-Al_2_O_3_ surface. The configuration of the PETN molecule corresponds to its relaxed position at the interface between (0001) α-Al_2_O_3_ and (**a**) (110) and (**b**) (101) PETN crystal surfaces. Bader charges localized on PETN molecules are also shown.

To study optical absorption of α-Al_2_O_3_-PETN composites, we started with performing electronic structure calculations of the pristine α-Al_2_O_3_ (0001) surface, surface containing oxygen vacancies and the α-Al_2_O_3_ (0001) surface with adsorbed PETN molecules. Taking into account that the electronic structure of an organic molecular crystal is nearly fully defined by the electronic structure of constituting molecules, we constructed simplified model supercells, which consist of an individual PETN molecule positioned at the (0001) α-Al_2_O_3_ surface (Figure 1). Similar model structures have been recently employed to investigate the photo-chemistry of the PETN-MgO interfaces [22] and interactions of various nitro-containing molecules with silicon [24], aluminum [25], and aluminum oxide [26] surfaces.

To simulate the Al_2_O_3_ surface we used a periodic slab model. Surface slab was cut from the bulk α-Al_2_O_3_ structure to form the surface with the (0001) orientation, with the supercell lattice vectors of *a* =14.288, *b* =16.499 Å, and *c* =26.169 Å. The vacuum layer of 20 Å placed on the top of the (0001) Al_2_O_3_ surface was intended to minimize interactions between supercells in ***z*** direction and to ensure that electronic states of different slabs do not overlap.

Further, individual PETN molecules were placed on an ideal (0001) α-Al_2_O_3_ surface (Figure 1). The orientation of the PETN molecule was chosen in such a way that it mimics the interface between (0001) α-Al_2_O_3_ and (110) and (101) low energy facets [27,28,29] of the PETN crystal (Figure 1a,b).

In the fully relaxed structural *configuration-1* (with the relative position of the PETN molecule *versus* the oxide surface corresponding to its orientation at the PETN crystal (110) surface; Figure 1a), oxygen atoms of nitro groups are located above the surface aluminum atoms. The calculted interatomic distances between O and surface Al atoms are 2.000 and 2.168 Å, respectively.

The orientation of the PETN molecule in the *configuration-2* (Figure 1b) corresponds to its orientation at the (101) surface of PETN crystal with only one C-CH_2_-O-NO_2_ tail of the PETN molecule directed almost perpendicualar to the surface. In this model, one of the O atoms of the NO_2_ group is located above the aluminum atom at the distance of 1.992 Å.

Because van der Waals interactions are important for the accurate determintion of adsorption/desorption energies, we included vDW corrections in our calculations. The calculated binding energy for the PETN molecule adsorbed on the α-Al_2_O_3_ surface in the *configuration-1* (1.59 eV) is two times higher than that obtained from the *configuration-2* (0.80 eV). A comparison of the adsorbtion energies obtained for α-Al_2_O_3_-PETN with those reported earlier for MgO-PETN system (0.43 eV) [22] indicates significantly stronger binding of the PETN molecule to Al_2_O_3_ than to MgO surface. This actually means that the Al_2_O_3_-PETN interface should be more stable than MgO-PETN.

#### 2.1.2. Optical Absorption of the Pristine α-Al_2_O_3_ (0001) Surface

**Figure 2:**
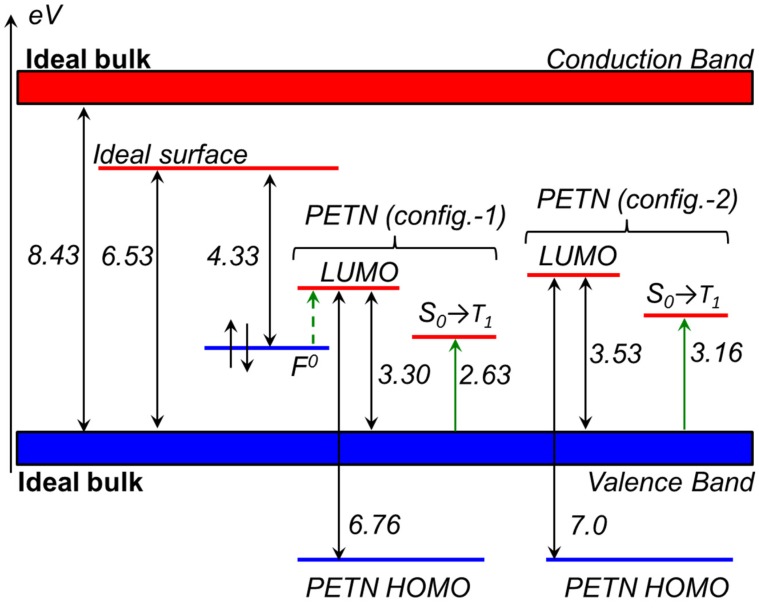
The relative energies of the (0001) α-Al_2_O_3_ surface, F^0^-center, and PETN- induced electronic states in the bulk band gap are shown. The dashed green arrow indicates the predicted F^0^-center (HOMO) → PETN (LUMO) transition with the energy of 1.2 eV. The solid green arrows correspond to singlet-triplet excitations from the aluminum oxide surface to PETN.

The optical and electronic properties of α-Al_2_O_3_ crystals have been previously studied experimentally and theoretically. Hence, our goal here is to reproduce all major features of the optical spectra and accurately interpret them before we can move on to the study of the composite system. The calculated band gap of an ideal α-Al_2_O_3_ bulk crystal (8.43 eV, Figure 2) is consistent with the experimentally measured gap (8.7 eV) [30] and shows better agreement than earlier estimations (7.2 [31], 7.77 [32] and 8.06 [31] eV). The obtained band gap of the (0001) α-Al_2_O_3_ surface is reduced to 6.53 eV (Figure 2), which is consistent with the earlier theoretical studies [31,32].

**Figure 3:**
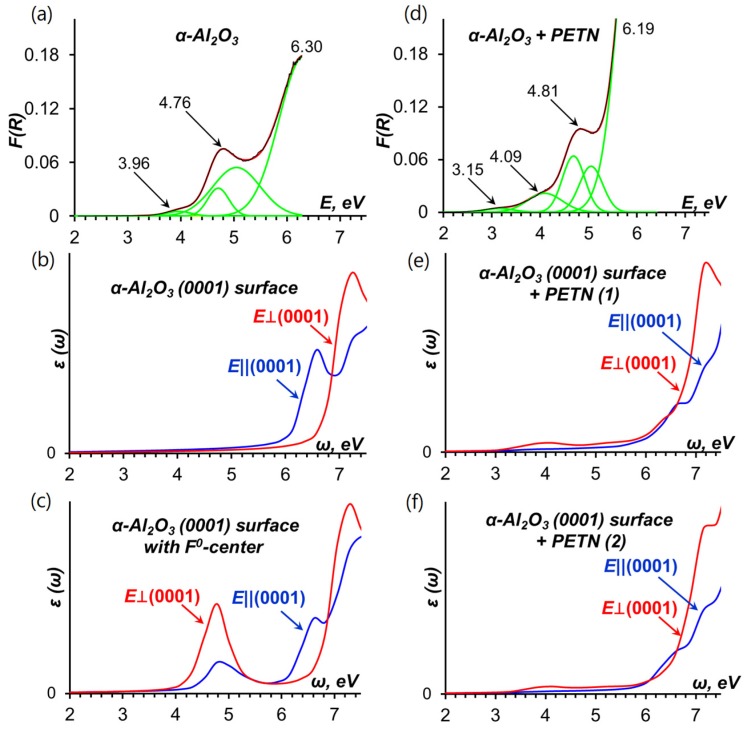
(**a**) Kubelka-Munk function of experimentally measured optical reflectance spectrum of α-Al_2_O_3_ (**black** line) is approximated with a set of Gaussians (**red** line); (**b**) Imaginary part of dielectric function of the pristine (0001) α-Al_2_O_3_ surface for light propagation parallel (**blue** line) and normal (**red** line) to the (0001) direction; (**c**) Imaginary part of dielectric function of the (0001) α-Al_2_O_3_ surface containing an F^0^-center for light propagation parallel (**blue** line) and normal (**red** line) to the (0001) direction; (**d**) Kubelka-Munk function of experimentally measured optical reflectance spectrum of the α-Al_2_O_3_-PETN composite (**black** line) is approximated with a set of Gaussians (**red** line); Imaginary part of dielectric function of (0001) α-Al_2_O_3_ surface with PETN molecule adsorbed in (**e**) configuration-1 and (**f**) configuration-2 for light propagation parallel (**blue** line) and normal (**red** line) to (0001) direction.

Figure 3 compares experimental and theoretically simulated spectra. The experimentally measured optical absorption spectrum of α-Al_2_O_3_ samples is depicted in Figure 3a and consists of two pronounced absorption bands, the first band with a well-defined maximum at 6.3 eV and the shoulder with a maximum at 4.76 eV. The energy of the intense transition at 6.3 eV agrees well with the calculated surface energy gap (6.53 eV, Figure 2) and therefore can be attributed to absorption of the α-Al_2_O_3_ surface. The calculated plot of frequency-dependent imaginary part of the dielectric function *ε*(ω) depicted in Figure 3b shows a distinct maxima at 6.58 and additionally confirms that this band is due to optical absorption of the pristine (0001) α-Al_2_O_3_ surface.

Since transitions in the range from 4.5 to 5.05 eV are most likely related to surface defects, we explicitly simulated an F^0^-center at the (0001) α-Al_2_O_3_ surface as oxygen vacancies are the most frequent defects on oxides [33]. We found that the F^0^-center generates an occupied state in the surface band gap. This state lies at about 2.20 eV (Figure 2) above the top of the valence band of the pristine α-Al_2_O_3_, which agrees well with the results of earlier theoretical study [34]. The energy gap between the F^0^-center-induced state and the top of the Al_2_O_3_ surface conduction band is 4.33 eV (Figure 2), which should roughly approximate the experimental peak, *i.e.*, ~ 4.76 eV (Figure 3a). The calculated optical absorption spectrum of the oxide surface with F^0^-center depicted in Figure 3c indicates a maximum at 4.78 eV, which is very close to the experimentally observed peak at 4.76 eV. Therefore, the shoulder in the experimentally measured spectrum of α-Al_2_O_3_ is related to the transitions associated with absorption of F^0^-centers [35].

In addition, we note that the lowest energy peak at 3.96 eV observed in the α-Al_2_O_3_ spectrum may be associated with absorption of Mn-ion impurity centers [36], which are typical for alumina materials.

These results illustrate a very close agreement between the measured and calculated optical parameters of the system under study as well as with previously published reports by other researchers. This correspondence serves as a solid indication of quality of this research and allows us to expect accurate conclusions related to the α-Al_2_O_3_-PETN composites.

#### 2.1.3. Optical Absorption of the α-Al_2_O_3_ (0001)-PETN Interface

The optical absorption of PETN molecules and crystals were recently studied in great detail [23]. The UV absorption spectrum of PETN consists of three broad bands: a strong electronic transition at or below 193.5 nm (>6.41 eV) [37,38,39] and two weak transitions at 260 nm (4.77 eV) [37,38] and 290 nm (4.27 eV) [37]. All three excitations are localized predominantly on the -NO_2_ groups of PETN [23]. The former two correspond to singlet-singlet transitions, whereas the last one was interpreted as a combination of two overlapping singlet-triplet transitions [23].

The diagram shown in Figure 2 indicates additional unoccupied states (LUMO) in the oxide band gap, which lie 3.30 and 3.53 eV above the top of the valence band. These states are localized on O-NO_2_ fragments of PETN molecule and formed from 2*p* atomic functions of oxygen and nitrogen atoms. The presence of the extra occupied states generated by the surface oxygen vacancy in the band gap of α-Al_2_O_3_ and of the unoccupied states attributed to PETN molecules adsorbed on (0001) α-Al_2_O_3_ surface (Figure 2) implies that additional optical excitations should be observed in the absorption spectrum of the oxide. Energetic considerations suggest that this transition should be characterized by the excitation energy of the order of 1.2 eV. We note that this expected excitation energy is very close to the laser excitation energy of 1.17 eV (the first harmonic). This prediction further guides our absorption measurements to validate the theory [40].

There is a clear difference between the measured α-Al_2_O_3_ spectrum shown in Figure 3a (with three obvious peaks with maximums at 6.3, 4.76, and 3.96 eV) and α-Al_2_O_3_-PETN composite spectrum depicted in Figure 3d (with four maximums at 6.19, 4.81, 4.09, and 3.15 eV). We note that the presence of PETN in the composite is reflected in two facts, intensity of the three of the existing bands becomes higher and one additional band appears in the spectra.

The calculated optical absorption spectra depicted in Figure 3b,c,e,f represent each distinguishable component of the α-Al_2_O_3_-PETN composite system. Hence, a comparative analysis of the experimental and simulated spectra suggests a reasonable interpretation of the absorption spectrum of the α-Al_2_O_3_-PETN composite.

The main high intensity absorption band at the energies >6 eV is related to the α-Al_2_O_3_ surface absorption. The F^0^-center absorbs light with energy of 4.78 eV (Figure 3c), which is consistent with earlier estimates (4.62 and 5.03 eV) [41] obtained from embedded cluster calculations using TD-DFT and CASPT2 methods. Hence, the energy range from 4.76 to 5.05 eV observed in both the pristine α-Al_2_O_3_ spectrum and the α-Al_2_O_3_-PETN composite spectrum is associated with the oxygen vacancy absorption.

Further, the broad low intensity band at 3.15 eV (Figure 3d) appears only in the spectrum of the composite but not in the pristine alumina. The energy maximum agrees well with the energy gaps of 3.30 and 3.53 eV and the corresponding singlet-triplet transitions at 2.63 and 3.16 eV, respectively (Figure 2), depending on the PETN configuration on the (0001) α-Al_2_O_3_ surface (Figure 1). This excitation is attributed to PETN absorption at the interface and corresponds to the transition of an electron from the alumina surface to the adsorbed PETN molecule, creating an excited state of PETN.

**Figure 4:**
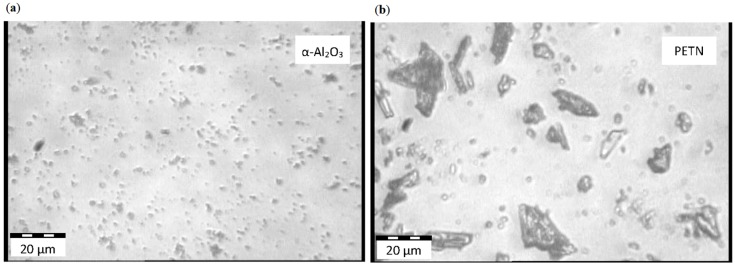
Microphotographs of (**a**) α-Al_2_O_3_ and (**b**) PETN samples.

Furthermore, while the 4.09 eV peak (Figure 3d) nearly coincides with 3.96 eV (Figure 3a) and may be attributed to the Mn-ion impurity’s absorption, the visible increase of the intensity and breadth of this peak suggests the existence of a new absorption band in this spectral range. The calculated spectra (Figure 3e,f) clearly show the broad absorption peak with the maximum at 4.0 eV while the model interfaces (Figure 4a,b) obviously did not include Mn impurities unlike experimental samples in which Mn is always present.

Consequently, we recall that absorption of PETN falls in the range of 3.88–6.5 eV [23] and it should be expected that this will contribute to the overall interface optical properties. For example, the energy, 4.27 eV [23], is associated with the two overlapping singlet-triplet transitions of the PETN molecule. This lends an additional support to the notion that the electronic excitations fully localized on PETN contribute to the absorption of the interface. Similarly, the two next PETN transitions at 4.77 eV contribute to the increased intensity of the 4.81 eV peak of the Al_2_O_3_-PETN interface. Therefore, the Al_2_O_3_-PETN interface has distinct optical electronic excitations that differ from individual components, alumina and PETN.

### 2.2. Decomposition of Charged and Excited PETN Molecules

The goal of this section is to determine whether decomposition of the PETN molecule from either its excited or charged state can be triggered by laser excitation energy of 1.17 or 2.3 eV and to compare this process to the ground state chemistry. In our consideration, the interactions between the adsorbed PETN molecule and the F^0^-center on the Al_2_O_3_ surface play a crucial role in initiation of such photochemical decomposition reactions. In the absence of solid-state calculations of PETN molecules decomposing on the oxygen deficient Al_2_O_3_-PETN interface, which is quite challenging, especially with hybrid functionals, we will make an attempt to interpret the obtained results in terms of previously performed modeling of the decomposition of isolated PETN molecules and ion radicals. We will analyze here activation barriers and reaction energies to link (or correlate) them with the characteristic excitation energies observed in the optical spectrum of our composite.

**Figure 5:**
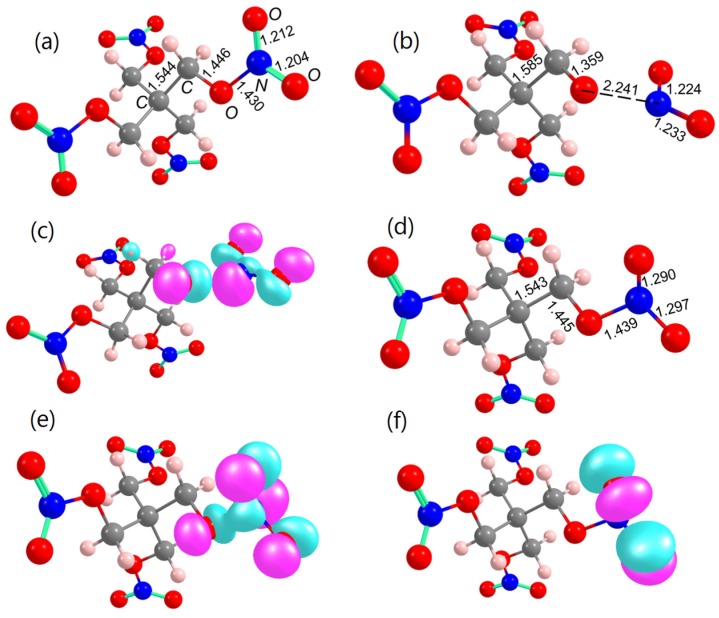
The geometric configurations of equilibrium molecular structures of (**a**) the ground state neutral PETN molecule; (**b**) PETN radical anion; (**c**) electron component of PETN radical anion; (**d**) PETN in the triplet state; (**e**) electron and (**f**) hole components of PETN in triplet state.

For the intended analysis, we simulated two different scenarios of the formation of the initial PETN state: (i) a PETN anion radical that can be formed due to charge transfer from the F^0^-center to PETN, similarly to the process observed for the MgO-PETN interface [22] and (ii) PETN in its triplet state that can occur due to the vertical HOMO-LUMO excitation (F^0^-center-PETN transition) with the energy of ~1.2 eV (Figure 2) or as a result of an electron excitation from the surface to the molecule observed at ~2.3 eV (Figure 2 and Figure 3). We limited our decomposition simulations to the O-NO_2_ (Equation (1)) bond homolysis only as it is the main decomposition pathway of the neutral ground state PETN (Figure 5a) in both gaseous and solid states (Equation (1)) and requires ~35 kcal/mol [29].

C_5_H_8_N_4_O_12_ → NO_2_ + C_5_H_8_N_3_O_10_ (1)

It was discovered that the similar process proceeding from the PETN radical anion state requires 50% lower energy (18.0 kcal/mol, Table 1) [22] than the ground state decomposition (~ 35 kcal/mol) [29]. An extra electron in the equilibrium structure of PETN radical anion (Figure 5b) is localized on the –ONO_2_ molecular fragment (Figure 5c) with the elongated O–N bond. Having high positive electron affinity, the PETN molecule readily traps an electron, gaining 2.4 eV (Table 1). We suggest that the formation of PETN anion radical is feasible at the Al_2_O_3_-PETN interface atop the F^0^-center through withdrawal of an electron from the vacancy. Once an electron is transferred to the PETN LUMO, the system will gain energy and the level will go down in energy, thus facilitating new transitions from and to this state. This will likely to trigger dissociation of PETN with a low activation barrier through the charged state potential surface.

The formation of the vertical singlet-triplet transition in PETN was simulated by using TD B3LYP and ΔSCF approximations. The energy required to form a triplet state is 3.88–4.22 eV, depending on the method [42]. The fully relaxed equilibrium structure of the triplet state PETN (Figure 5d) lies 2.75 eV above the ground state equilibrium structure. The high relaxation energy indicates that the exciton is tightly bound. Both the electron and hole components of the triplet state are well-localized on the distorted -O-NO_2_ fragment (Figure 5e,f) with the elongated by ~0.07–0.09 Å N-O bonds of the nitro group as compared to the neutral molecule (Figure 5a). The O-NO_2_ bond cleavage of the PETN molecule in its triplet state requires only 4.5 kcal/mol, and the reaction proceeds with the energy release of 28.7 kcal/mol (Table 1).

These simple estimates vividly illustrate that decomposition of PETN on the Al_2_O_3_-PETN interface can be triggered with a very low energy, significantly lower than the ground state decomposition reaction. However, the formation of the excited state (via direct vertical HOMO-LUMO excitation fully localized on PETN) or charged PETN state (via electron transfer from the surface to PETN) on the otherwise ideal interface would require ~ 3–4 eV. This high energy is inconsistent with either first (1.17 eV) or second (2.33 eV) harmonic laser excitation energies. On the other hand, Figure 2 clearly indicates that the transition from F^0^-center (HOMO) to PETN (LUMO) at the interface would have an excitation energy ~1.2 eV, close to the first harmonic 1.17 eV. The singlet-triplet excitation associated with the electronic transition from the surface HOMO to PETN LUMO requires only 2.63 eV, close to the second harmonic 2.33 eV. The charge transfer from the oxygen vacancy, which typically traps nearly two electrons on oxides, to PETN is energetically favorable. This situation is, indeed, somewhat similar to the MgO-PETN interface. We therefore suggest that the interactions of oxygen vacancies and the PETN molecules at the Al_2_O_3_-PETN interface would facilitate the formation of charged and/or excited PETN states that would consequently rapidly dissociate, producing NO_2_ and will be accompanied by heat release.

## 3. Methods

### 3.1. Details of Calculations

Solid state periodic calculations were performed by employing density functional theory (DFT) [43,44] with vDW-DF [45,46,47] functional of Langreth, Lundqvist *et al.* which includes corrections of van der Waals interactions as implemented in the VASP code [48,49,50]. To correct the significantly underestimated band gap energies, obtained from vDW-DF, a self-consistent single point calculation was performed for each configuration by using hybrid PBE0 functional [51]. The projector augmented-wave (PAW) pseudo-potentials [52] were used.

In calculations of an ideal Al_2_O_3_ crystal, the convergence criterion for total energy was set to 10^−5^ eV, and the maximum force acting on each atom in the periodic cell was set not to exceed 0.02 eV/Å. We used 4 × 4 × 2 Monkhorst-Pack *k*-point mesh, and the kinetic energy cut-off was set to 520 eV. The calculated lattice constant of the hexagonal unit cell, *a* = 4.763 Å, *c* = 12.985 Å, agrees with the experimental lattice vectors of *a* = 4.757 Å, *c* = 12.9877 Å [53] within 0.1%.

Optical absorption spectra were obtained by calculating the frequency-dependent imaginary part of the dielectric function [54] within VASP.

Gas-phase fragmentation pathways of ionized and excited PETN molecules were studied using Becke three-parameter hybrid B3LYP [55,56] functional with 6-31+G(d,p) basis set within Gaussian 09 program [57].

### 3.2. Details of Experiment

In this work, we used chromatographic alumina as a mixture of α- and θ-phases. The phase composition was determined by the diffractometer DR-02 RADIAN (NTC Expert center, Moscow, Russia), with a wavelength λ = 1.541874 Å. In order to obtain the pure α-phase aluminum oxide, the initial sample was subjected to a heat treatment in a muffle furnace in the air at temperature 1300 °C for 2 h, followed by slow cooling. Ground oxide powder was manually ground in an agate mortar, the final powder grain size was ~1 μm (Figure 4). Al_2_O_3_-PETN composite particles (Figure 4) with the 0.5% weight concentration of PETN were prepared through the following procedures. Samples of PETN were initially grinded in agate mortar. Al_2_O_3_ powder was subsequently added to the mortar and two powders have being mixed for 5 min. The mixture was heated in the drying oven for 10 min at temperature 144 °C to allow PETN to melt and cover uniformly Al_2_O_3_ particles. The selected oven temperature provided a guaranteed meltdown of PETN grains weighting ~3.2 g in composite in 10 min as the PETN melting point is 141.3 °C (see, e.g., [58]).

**Figure 6:**
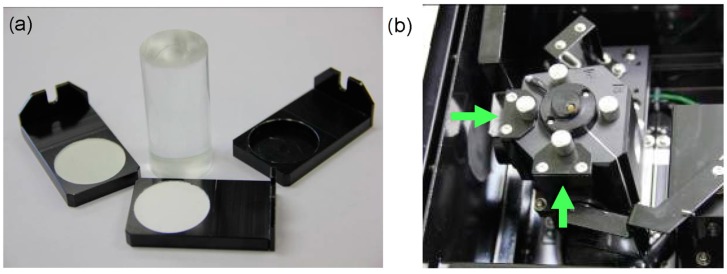
The photo of (**a**) the measuring cell and the glass cylinder used to form tablets and (**b**) the UV-VIS-NIR integrating sphere attachment ISR-3100 with the installed measuring cells.

The optical reflectance spectra of the obtained composite were measured and recorded in the range 190 to 1200 nm (1.03–6.52 eV) using Shimadzu UV-3600 spectrophotometer (Shimadzu Corporation, Kyoto, Japan) with UV-VIS-NIR integrating sphere attachment ISR-3100. Samples taken for the study were weakly pressed tablets formed in a glass cylinder with the recess of the measuring cell (Figure 6). Optical reflectance spectra of pure aluminum oxide, pure PETN, and the composite have been registered with respect to barium sulfate powder (the basic instrument line was built for two samples of barium sulfate, one of which is then changed to the sample under study). The obtained dependences were then transformed using the Kubelka-Munk formula.

## 4. Summary and Conclusions

Our research was inspired by recent experiments on laser initiation of explosive decomposition of PETN-MgO mixtures [18,19,20,21]. The current project aimed at ascertaining the ability of the commonly used Nd:YAG lasers to trigger the decomposition chemistry of α-Al_2_O_3_-PETN composites by using the first (1.17 eV) or second (2.33 eV) harmonic irradiation. In our study, we combined the experimental measurements of optical absorption spectra and the electronic structure calculations of the α-Al_2_O_3_-PETN composite as well as first principles modeling of decomposition of PETN anion radicals and excitons.

With the expectation for adsorption of PETN on highly ionic MgO to differ from adsorption on partly-covalent corundum (Al_2_O_3_), it is interesting to establish that oxygen vacancies play a crucial role in photochemistry of both oxide-energetic material interfaces. The different character of chemical bonding in MgO and Al_2_O_3_ is manifested in adsorption of PETN on the surfaces and the corresponding binding energies of interfaces. Indeed, the physical adsorption of the PETN molecule on the ideal (001) MgO surface is characterized by a low binding energy of 0.43 eV, while the vacancy serves to cause much stronger chemical adsorption and increases the binding energy more than three times to >1.2 eV [22]. This implies that PETN molecules are attracted to oxygen vacancies on ionic MgO surface due to Coulomb interactions. While the electrostatics at the Al_2_O_3_-PETN interfaces work the same way, the PETN molecules strongly adsorb even on the pristine ion-covalent Al_2_O_3_ surface with the binding energy of 1.59 eV (see Section 2.1). It is natural to expect that the binding between the F^0^-center of corundum and the molecule is also strong.

Quantum-chemical calculations show that the electronic structure of the α-Al_2_O_3_-PETN composite is significantly different from the structures of individual components, corundum, and PETN. In particular, the top of the valence band of the interface is formed by the oxygen wave functions of the α-Al_2_O_3_ (0001) surface. The bottom of the conduction band is composed from the molecular orbitals of PETN-O-NO_2_ groups. The obtained optical band gap of the α-Al_2_O_3_-PETN composite, ~ 3.3–3.6 eV, is almost two-times lower than the band gap of the ideal α-Al_2_O_3_ (0001) surface, 6.53 eV, and by far lower than the band gap of the ideal bulk corundum crystal, 8.43 eV. In excellent agreement with theory, the measurements indicate that the α-Al_2_O_3_-PETN composite starts absorbing light with the energy as low as ~2.6 eV. The distinct peaks in the optical absorption spectrum are attributed to F^0^-centers and electronic transitions from the α-Al_2_O_3_ (0001) surface to PETN.

Further, the relative positions of the F^0^-center state and the PETN associated states predict an optical transition with the energy of ~1.2 eV, which is very close to the first harmonic laser irradiation energy, 1.17 eV. Additionally, the formation of the PETN excited triplet state requires 2.63 eV, which is close to the second harmonic laser irradiation energy of 2.3 eV. Similarly to the PETN-MgO interfaces, F^0^-centers will likely to donate an electron to PETN due to high positive electronic affinity of the latter.

Furthermore, the analysis of fragmentation pathways suggests that much lower activation barriers are required to trigger the O–NO_2_ bond dissociation of the negatively charged and excited PETN molecules in comparison to the neutral ground state molecule decomposition barriers. Thus, the NO_2_ loss of the neutral ground state PETN molecule requires 35 kcal/mol [29] while the PETN radical anion requires two-times less energy, 18.0 kcal/mol. The same reaction needs only a negligible energy, 4.5 kcal/mol, if initiated from PETN in the triplet state, and proceeds exothermically releasing 28.7 kcal/mol of heat.

These results predict that photodecomposition of PETN molecules from the α-Al_2_O_3_-PETN composites can be reliably initiated with Nd:YAG laser excitations of 1.17 and 2.33 eV. The light absorption of this system is mainly defined by the band alignment between the energetic material and metal oxide at their interface. This alignment depends on the choice of a wide band gap oxide substrate, which provides a unique possibility to tune up the laser light frequency at which the photoinitiation process is most efficient. The control over the laser initiation of these materials could solve many important problems of practical applications related to security, safety, and environmental sustainability.

## Figures and Tables

**Table 1:** The decomposition of PETN in its equilibrium, [PETN], charged, (PETN)^-^, and excited, (PETN)*, states is illustrated by the corresponding energy of the initial state formation (in eV), activation barrier for the O-NO_2_ homolysis reaction (in kcal/mol), and the reaction energy (in kcal/mol).

Initial State	Formation Energy	Activation Barrier	Reaction Energy
[PETN]	0	35.0	35.0
(PETN)^-^	−2.4 (*EA* of isolated molecule)	18.0	18.0
(PETN)*	3.88–4.22 (Vertical excitation)	4.5	−28.7
